# Bow-legs

**Published:** 1893-09

**Authors:** 


Bow-Legs.—Dr. A. E. Hoadley (*Chicago Clinical Review*) says: Rugged and rapid development produce bow-legs, and more commonly straight legs, which will uniformly correct themselves without assistance.

The severe forms of ordinary bow-legs, especially where the joint itself partakes largely of the deformity, will require treatment by restraining and corrective force.

The prognosis, in the ordinary forms of bow-legs, is very favorable under the influence of mild corrective force.

The prognosis in rachitic bow-legs is unfavorable. When this condition is of long standing, it is practically not amenable to treatment by gradual corrective force, and, therefore, should be corrected by osteotomy. The rachitis itself requires the most careful and comprehensive constitutional treatment.

The anatomical arrangement of muscles favors the spontaneous correction of bow-legs, and the biceps is the most important in the exercise of this corrective force. In the opposite condition, or knock-knee, there are no opposing muscles that can act as correctors of the deformity.

The strong contrast between these two conditions, bow-legs with a tendency to recovery, and knock-knee with a tendency to progression and difficulty of correction, is due entirely to the anatomical arrangement of the muscles.

